# Current Status of Ovarian and Endometrial Biomarkers in Predicting ART Outcomes

**DOI:** 10.3390/jcm13133739

**Published:** 2024-06-26

**Authors:** Michelle Volovsky, David B. Seifer

**Affiliations:** Department of Obstetrics, Gynecology and Reproductive Sciences, Yale School of Medicine, New Haven, CT 06510, USA; david.seifer@yale.edu

**Keywords:** biomarkers, ovarian, endometrial, ART, predictor, outcomes

## Abstract

This review evaluates the role of ovarian and endometrial biomarkers in predicting outcomes in assisted reproductive technology (ART). It highlights established ovarian biomarkers such as the anti-Müllerian hormone (AMH) and follicle-stimulating hormone (FSH), alongside emerging ones like growth differentiation factor 9 (GDF9), bone morphogenetic protein 15 (BMP15), connexin, and granulosa cell gene profiles. Additionally, the paper explores endometrial biomarkers such as ERA, BCL6, and immune markers, as well as the potential for genomic and proteomic technologies in customizing implantation. It concludes that while many of these biomarkers show promise, their clinical integration requires rigorous research and validation to confirm their safety and utility in ART.

## 1. Introduction

Biomarkers serve as quantifiable biological indicators, signaling various medical conditions or states within an organism. These molecular beacons are instrumental for the early detection of a dynamic physiologic state or disease, tracking the efficacy of treatment and potentially predicting medical outcomes. They are part of a larger endeavor of precision or personalized medicine, which strives to optimize diagnostic testing and therapy for each individual patient. In assisted reproductive technologies (ART), the discovery of dependable biomarkers related to ovarian and endometrial function is pivotal in elevating ART procedure successes, with the aspiration of ultimately leading to the birth of a healthy child. Predictions of ovarian response and endometrial receptivity are informative to setting realistic patient and physician expectations, tailoring treatment approaches, reducing complications, and elevating the success rates of ART treatment.

This narrative review aims to explore the existing scope of the female contribution to fertility, examining ovarian and endometrial biomarkers in their respective roles of their anticipation of ART outcomes. We include both traditional and possible emerging biomarkers with discussion of both serum and cellular markers. While serum ovarian biomarkers provide readily accessible methods through venipuncture, we include granulosa cell (GC) analysis because it may ultimately reveal future noninvasive ovarian biomarkers that are currently not realized. Furthermore, future technological advancements may facilitate easier access to GCs, potentially rendering GC analysis a more viable option.

In tandem with ovarian function, the endometrial environment is vital in completing the fertility puzzle. The endometrium’s receptivity, a critical determinant for successful implantation, is being increasingly deciphered by various biomarkers and genetic expression profiling. This review will discuss the applications of emerging research that are likely to complement our understanding of endometrial receptivity and ultimately implantation. Together, ovarian and endometrial biomarkers have the potential to refine fertility treatments, ushering in an era of targeted therapy and improved prognostic capabilities in reproductive medicine.

### Search Strategy

We conducted a thorough review of the literature, focusing on ovarian and endometrial biomarkers and their application in ART. The primary databases for our search included PubMed, Medline, the Cochrane Library, and Google Scholar. Our initial search was broad, encompassing the terms ‘ovarian’, ‘endometrial’, ‘marker’, ‘biomarker’, ‘IVF’, ‘ART’, ‘outcomes’, and ‘predictor.’ The search was aimed at identifying relevant biomarkers for each category—ovarian and endometrial. Once these biomarkers were identified, we executed a more focused search for each specific one. This subsequent phase of the literature review involved detailed search’s using general terms associated with biomarkers, including ‘ART’, ‘IVF’, ‘outcomes’, ‘predictor’, and ‘marker’, as well as the individual biomarkers themselves, specifically ‘AMH’, ‘FSH’, ‘LH’, ‘CA-125’, ‘GDF9’, and ‘BMP15’. Each potential marker was investigated in connection with ART outcomes to consolidate existing knowledge and identify gaps in current research.

The review included literature published in the English language concerning both cellular and circulating biomarkers in human subjects. The selected studies encompassed a range of research designs, from retrospective analyses to prospective observational studies and meta-analyses. Each study was evaluated for its contribution to the understanding of ovarian or endometrial biomarkers and their correlation with ART success rates.

## 2. Ovarian Biomarkers

### 2.1. Conventional Biomarkers of Ovarian Function

#### 2.1.1. Anti-Mullerian Hormone (AMH)

Anti-Müllerian hormone (AMH) is considered one of the key biomarkers for predicting ovarian response during ART. While it may not be the sole biomarker used in this context, it has evolved to be one of the earliest and most reliable due to its relatively stable levels throughout the menstrual cycle and its direct correlation with the quantity of preantral and antral follicles. Thus, AMH is a fundamental component of the ovarian reserve assessment and is used widely worldwide in fertility clinics to tailor individual patient ovulation induction treatment plans, predict outcomes for ART and set realistic expectations for both patients and their treating physicians.

Seifer et al. were the first to demonstrate AMH as a predictive marker for ovarian response in ART cycles [1]. Analyzing day 3 serum samples, they revealed a more than 2.5-fold difference in AMH levels between women who retrieved a higher number of oocytes compared to those with fewer, asserting a robust association between higher serum AMH levels and oocyte yield. Muttukrishna et al. found that AMH was a more reliable marker for oocyte retrieval than follicle stimulating hormone (FSH) and inhibin B [2]. An example of another early study that followed identified lower pregnancy rates in IVF cycles for women with reduced AMH levels [3].

Eventually, a meta-analysis compared AMH’s accuracy with antral follicle count (AFC) in predicting poor ovarian response and post-IVF nonpregnancy. The study proposed AMH as a viable alternative or adjunct to AFC, given its objectiveness and accessibility via venipuncture compared to AFC’s reliance on ultrasonography, an ultrasonographer and its associated variables of interpretation and patient convenience [4]. Similarly, Majumder et al. found that both AMH and AFC strongly correlated with the quantity and quality of oocytes and embryos [5].

In conjunction with these analyses, the systemic review by La Marca and colleagues also underscored the practical advantages of AMH and reinforced the finding that AMH was found to be a better predictor of ovarian response for controlled ovarian stimulation than patient age, FSH, estradiol, or inhibin B [6]. Subsequently, a large systematic review and meta-analysis comprising 5373 women investigated whether serum levels of AMH could predict implantation and clinical pregnancy in women undergoing ART [7]. This study reported a diagnostic odds ratio (OR) for AMH as a predictor of implantation in women with unspecified ovarian reserve of 1.83 (95% CI 1.49–2.25) and an OR for clinical pregnancy of 2.10 (95% CI 1.82–2.41), indicating a weak yet present association with ART outcomes. However, AMH did not significantly predict outcomes in women with polycystic ovary syndrome (PCOS), having an OR of 1.18 (95% CI 0.53–2.62).

Traditional stimulation protocols were compared with new AMH patient-based tailored protocols in a study of 769 women [8]. The introduction of AMH-tailored protocols led to a significant increase in embryo transfer rates, pregnancy rates and live birth rates as well as a notable reduction in ovarian hyperstimulation syndrome incidence. Furthermore, Vural et al. aimed to establish cut-off values for AMH in predicting ovarian response, discovering that lower AMH levels correlated with a lower number of retrieved oocytes, independent of age and FSH and LH levels, indicating AMH and AFC as superior predictive markers [9].

The impact of ultralow AMH levels on ART outcomes was evaluated in a retrospective analysis that included 5087 fresh ART cycles and 243 thawed cycles, specifically identifying those with AMH levels at or below 0.16 ng/mL (the lowest known positive threshold level using the most widespread assay at the time, the Beckman–Coulter AMH test) [10]. Their findings revealed a high total cancellation rate of 54% for fresh cycles, concluding it is crucial to counsel patients on the likelihood of cycle cancellation and the potential for poor outcomes associated with such very low AMH levels while, however, highlighting that undetectable levels of AMH did not specifically mean sterility, as some women did conceive. However, it should also be underlined that a very low value at a young age is different from the same at an advanced age, and those who are younger still likely have a reasonable chance of pregnancy [11].

A large analysis of data from the Society for Assisted Reproductive Technology Clinic Outcome Reporting System (SART CORS) database focused on AMH levels and cumulative live birth rates (CLBRs) among women with diminished ovarian reserve, which represented 25.9% of the 133,442 total autologous retrieval cycles in the database [12]. The findings indicated a strong association between AMH levels and the number of oocytes retrieved, the number of embryos cryopreserved, the mean number of cumulative embryos transferred, and the percentage of cycles resulting in an embryo transfer. Multiple logistic regression analysis demonstrated that AMH is an independent predictor of CLBR, indicating a 39% increase in the likelihood of live birth with each unit (ng/mL) increase in AMH.

Additionally, age-specific analysis of AMH levels could offer a more accurate assessment of ovarian reserve and IVF success potential. A retrospective study of 17,120 women found that both median and mean AMH levels decreased progressively with age, which could further refine personalized approaches to fertility treatments [13]. Other studies have corroborated these findings. In a study of 1015 fertile women, AMH levels demonstrated a non-linear decline with age across various percentiles [14]. Another retrospective analysis of 2741 patients also revealed an age-dependent distribution of AMH levels [15]. Furthermore, AFC and AMH showed declines with age while FSH increased, with serum AMH and AFC starting to decline between ages 34 and 35, earlier than changes in FSH were observed [16].

Clinical studies have consistently illustrated AMH’s role as a pivotal early biomarker in predicting ovarian reserve, response to stimulation, and potential ART outcomes, and it is incorporated into the fundamental patient work up of infertility in clinical practice [17].

#### 2.1.2. Follicle Stimulating Hormone (FSH)

FSH, produced by the pituitary gland, plays key roles in regulating the menstrual cycle, including the growth and maturation of ovarian follicles.

Laying the groundwork for understanding the significance of early follicular phase day 3 FSH levels was the purpose of Scott et al.’s 1989 study [18]. By measuring FSH, LH, and estradiol on cycle day 3 across 441 patients and 758 IVF cycles, they established a clear stratification of pregnancy success rates based on these hormonal thresholds. Their findings demonstrated that lower day 3 FSH levels (<15 mIU/mL) were correlated with higher rates of pregnancy per IVF attempt, as compared to those with moderate (15 to 24.9 mIU/mL) or high (>25 mIU/mL) levels.

Expanding on this knowledge, a larger study involving 1868 cycles concluded that consistent day 3 FSH levels over 20 mIU/mL were inversely related to pregnancy achievement. Those with transient elevations saw reduced success, and recurrent high levels typically resulted in non-pregnancies, prompting recommendations against continued IVF attempts for such cases [19]. Additionally, Ashrafi et al. highlighted the prognostic value of basal FSH in a sample of 212 IVF cycles. Women with higher FSH levels (≥15 IU/mL) on cycle day 3 saw fewer follicles and oocytes retrieved, fewer embryos transferred, and a higher rate of cycle cancellation. This contrasted with women with lower FSH levels, who had more favorable outcomes, reinforcing day 3 serum FSH as a predictive marker for ovarian response [20].

Concurrent studies by Jun et al. and Loutradis et al. explored the effect of FSH receptor (FSHR) gene polymorphisms on IVF outcomes. Jun et al. linked the Ser/Ser genotype to a diminished ovarian response and lower clinical pregnancy rates, while Loutradis et al. found that the Asn/Ser genotype correlated with a more favorable response, reflected in an increased number of follicles and oocytes. These findings suggest that FSHR gene polymorphisms could also contribute to IVF outcomes [21,22]. Another study by Baldini et al. investigated the reason behind a lower-than-predicted number of cumulus oocyte complexes collected after controlled ovarian stimulation. The researchers hypothesized that this deficient ovarian response is associated with a single-nucleotide polymorphism, Asn680Ser, of the FSH receptor. Two groups of patients were studied: those with normal responses (n = 36) and those with abnormal responses (n = 31). Genetic assessments showed a significant prevalence of this polymorphism on the FSH receptor in the abnormal response group compared to the normal response group (*p* < 0.05), concluding that genetic evaluation of the FSH receptor, specifically for the Asn680Ser polymorphism, could be of predictive value for patients undergoing ART [23].

A retrospective study further assessed IVF eligibility criteria, focusing on women with baseline day 3 FSH levels ≥ 20 mIU/mL, which historically would suggest diminished chances. Analyzing 291 women over 482 cycles found a live birth rate of 8.6% per woman and 6% per cycle, thus challenging the notion that high baseline FSH levels alone should disqualify patients from IVF treatment up to the age of 45 [24].

Not only are high FSH levels potentially predictive of poor outcomes, but very low FSH levels can also signify reduced fertility. Very low FSH can indicate hypogonadotropic hypogonadism, where deficiencies in LH and FSH impair gametogenesis and gonadal steroid production. In women, this deficiency, marked by low gonadotropin and estradiol levels, varies in cause. While the reduced production of LH and FSH is well studied, reduced action is less explored. Molecular characteristics, signaling issues, aging, and polymorphisms can hinder gonadotropin action, crucial in medically assisted reproduction. These factors may cause resistance and hypo-response to ovarian stimulation. Understanding these issues can potentially improve outcomes for patients, especially those of advanced maternal age and hypo-responders, who may benefit from recombinant human FSH and LH treatment [25].

These studies reinforce the relationship between FSH levels and fertility outcomes. In clinical practice, a randomly timed AMH is an earlier and more sensitive biomarker of reproductive aging, preceding the temporal elevation in FSH levels. Together, AMH and FSH may provide a comprehensive picture of a woman’s fertility status, aiding in tailoring individual treatment plans and managing expectations for success.

### 2.2. Possible Emerging Ovarian Biomarkers for Future Clinical Use

#### 2.2.1. Growth Differentiation Factor 9 (GDF9) and Bone Morphogenetic Protein 15 (BMP15)

Alongside well-established serum markers such as AMH and FSH, other potential biomarkers of ovarian function have emerged. Growth differentiation factor 9 (GDF9) and bone morphogenetic protein 15 (BMP15) are essential paracrine regulators in female fertility, playing crucial roles in folliculogenesis. Thus, their relationship to IVF outcomes has been explored. Li et al. analyzed cells from 2426 cumulus–oocyte complexes and found that higher GDF9 and BMP15 mRNA levels correlated with successful pregnancy indicators such as oocyte maturation and fertilization rates, highlighting their association with embryo quality and IVF success [26].

Subsequent studies have investigated the stability and presence of these biomarkers in various reproductive conditions. Riepsamen et al. identified that GDF9 and BMP15 serum levels exhibited significant individual variability but remained relatively stable throughout the menstrual cycle, suggesting that these markers could reflect intrinsic ovarian activity [27,28]. Further research by the same group indicated that GDF9 and BMP15 serum levels corresponded with ovarian volume and antral follicle count, but not necessarily with PCOS-specific androgenic or metabolic symptoms [29].

In the context of endometriosis, a case-control study found that serum GDF9 and BMP15 levels did not significantly differ between endometriosis patients and controls, implying that the presence of endometriosis might not influence the serum levels of these markers, thus maintaining their promise in reproductive medicine applications [30]. Another study assessed GDF9 and BMP15 in the follicular fluid and GCs of IVF patients with a low prognosis, revealing that reduced levels in both follicular fluid and GCs were associated with lower live birth rates and higher cycle cancellation rates [31].

Although more clinical research is needed, the amalgamation of these studies supports the continued investigation of the utility of GDF9 and BMP15 as ovarian biomarkers.

#### 2.2.2. Connexin 43

Gap junctions, particularly those involving connexin 43 (Cx43), are integral to oocyte development and subsequent embryo viability post-IVF due to their potential role in oocyte–cumulus cell (CC) two-way communication. Tsai and colleagues delved into the correlation between mRNA levels of connexins (Cx37, Cx43, Cx45) and oocyte outcomes in 91 luteinized follicles from hyperstimulated ovaries. Their study revealed that a high expression of Cx43 was linked with favorable oocyte prognosis [32]. Thus, they proposed Cx43 mRNA levels as potential indicators for oocyte quality prediction.

However, research outcomes on Cx43’s role have been inconsistent. Hasegawa et al. found no significant correlation between Cx43 expression and fertilization or embryo cleavage rates and observed that lower Cx43 levels were present in CCS from higher-quality embryos [33]. Contrarily, Wang et al. observed a positive association between its levels and both junctional conductance and embryo quality post-IVF, suggesting that higher Cx43 expression might predict better pregnancy outcomes [34].

Exploring a different perspective, the influence of retinoids on oocyte fertilization capacity was examined with a focus on all-trans retinoic acid (ATRA). It was found that ATRA prompted rapid dephosphorylation of Cx43, potentially improving gap junction functionality and influencing oocyte competence [35]. Additionally, a separate study highlighted that elevated follicular fluid retinoic acid (RA) concentrations correlated with superior embryo quality. This evidence suggested that RA can modulate Cx43 expression, thereby affecting oocyte competence. It was hypothesized that retinoid levels in CCs could serve as a non-invasive measure for choosing viable oocytes for IVF and that modulating Cx43 with retinoids might enhance outcomes, especially in scenarios where implantation is problematic or conditions such as endometriosis are present [36].

While some findings suggest that increased levels of Cx43 correlate with better oocyte outcomes, others report no clear link or present conflicting data; thus, there is a need for further research to determine Cx43’s actual predictive value for IVF success.

#### 2.2.3. Granulosa Cell (GC) Gene Expression Profiles

While the analysis of GC gene profiles offers promising insights for predicting IVF outcomes, the practicality of such methods is currently limited in clinical settings. Despite these challenges, there is potential for GC analysis to become more accessible in the near future with numerous studies exploring its role as a biomarker for ART outcomes.

In an early exploration, McKenzie et al. investigated the gene expression of cumulus GCs in relation to embryo development [37]. In a cohort of eight IVF patients, the study identified a correlation between higher expression of HAS2, PTGS2, and GREM1 in CCs and the development of higher-quality embryos. A subsequent retrospective analysis assessed CC gene expression in relation to oocyte quality, identifying mRNA expression of VCAN and PTGS2 as potential markers of embryos with higher developmental potential [38]. In a larger study of 308 samples, Li et al. analyzed the gene expression of CCs in connection with oocyte maturity and fertilization success, noting distinct patterns in expression levels of genes like GJA1, SERPINE2, and PRSS35 that correlated with oocyte and embryo development stages [39].

Meanwhile, Papler et al.’s microarray analysis attempted to pinpoint gene expression signatures in GCs predictive of successful embryo implantation and fertilization but did not establish significant biomarkers for IVF outcomes [40]. Massoud et al.’s systematic review synthesized findings from 42 studies, which collectively highlighted a consistent link between CC genes involved in various biological pathways, including calcium homeostasis and glucose metabolism, with the quality of oocytes and embryos and subsequent IVF success [41].

Taken together, these studies represent a growing body of research focused on the potential of genetic markers in GCs to predict IVF success, reflecting the field’s progression towards the practice of more personalized and precise reproductive therapy.

#### 2.2.4. Other Potential Ovarian Biomarkers (Caspases, CA-125, BCL-6, PTEN)

Apoptotic biomarkers caspase-3 and caspase-7, and their inhibitor survivin, are vital in the process of programmed cell death and have been investigated as indicators of embryo quality in ART. Salehi et al. examined these biomarkers, including survivin, in cumulus cells from PCOS patients undergoing ART [42]. Findings revealed lower survivin levels and higher levels of caspase-3 and -7 in PCOS patients than in normal women. Lobach et al.’s research established a negative correlation between caspase-3 gene expression in human luteinized GCs and various outcomes of induced ovulation, such as pre-ovulatory follicle count and oocyte maturation, implicating higher caspase-3 expression with poorer ovarian response during IVF [43].

CA-125, a glycoprotein, has been examined for its potential to predict IVF outcomes with mixed results. Some studies have observed that CA-125 levels either on the day of hCG administration or post oocyte retrieval were correlated with pregnancy rates [44,45,46]. In contrast, other research has reported no prognostic significance of CA-125 levels for IVF outcomes [47,48]. Although one study that corroborated that CA-125 levels were higher in pregnant patients, they found no differentiation for ongoing pregnancies, undermining CA-125’s predictive ability for pregnancy outcomes [49]. While there is some evidence to suggest that CA-125 levels may rise in conjunction with pregnancy, its reliability and utility as a stand-alone predictor of IVF/ET success are consistently weak.

The potential link between ovarian inflammation, as indicated by BCL-6 expression, and pregnancy success [50] has been investigated. High BCL-6 expression was linked to lower pregnancy success. This study also noted racial disparities in BCL-6 gene expression levels, with non-white participants exhibiting higher levels. Interestingly, no significant correlation was found between BCL-6 expression in the endometrium and the ovary, suggesting that inflammation in these two reproductive sites may be independently regulated.

Recent studies conducted by Yao et al. in 2021 and 2022 investigated the expression of the PTEN gene, associated with cell death processes, in GCs [51,52]. The 2021 study found a slight decrease in PTEN expression in those who achieved pregnancy, while the 2022 study observed significantly lower PTEN mRNA levels in CCs around mature oocytes compared to immature ones. These insights suggest that PTEN expression analysis in GCs could be a possible avenue for enhancing the assessment of oocyte viability and predicting IVF success.

### 2.3. Future Directives

Moving beyond the traditional markers of AMH and early follicular FSH, research now delves into a more nuanced and individualized approach, considering the complex interplay of factors affecting fertility. The study of biomarkers such as GDF9, BMP15, and Cx43 may offer more promise on the multifaceted nature of ovarian response, embryo development, and successful pregnancy outcomes. With advancements in technology, GC gene profiles are likely to become more accessible, offering a promising complement to serum biomarkers. This shift could elevate the personalization of treatment strategies, as these profiles may eventually stand alongside AMH and early follicular FSH in guiding clinical decisions. The future of IVF could involve a sophisticated interplay between established serum hormone levels and emerging cellular genetic profiles. Additionally, the integration of artificial intelligence (AI) could revolutionize fertility treatments by analyzing vast datasets to identify patterns and predict outcomes more accurately, further enhancing the personalization and effectiveness of ART. AI can also aid in the analysis of potential ovarian biomarkers by efficiently processing and interpreting large datasets, identifying subtle patterns and correlations among the aforementioned biomarkers, leading to more precise predictions of ovarian response and treatment outcomes.

## 3. Endometrial Biomarkers

### 3.1. Endometrial Receptivity Array (ERA)

Alongside insights into ovarian function, understanding the endometrial environment is believed to be as important for achieving successful pregnancy. In the quest to optimize implantation, and thus reproductive outcomes, the endometrial receptivity array (ERA) initially emerged as a potentially groundbreaking tool. Pioneered by Díaz-Gimeno et al., it was said to offer a genomic approach to enhancing the synchronization between embryo transfer and an individual’s unique window of implantation (WOI) [53]. By analyzing cohorts of endometrial samples, this study identified a transcriptomic signature of 134 genes and demonstrated high specificity and sensitivity for both endometrial dating and pathological classification, proposing that this diagnostic tool has clinical utility in reproductive medicine.

Following these initial findings, Ruiz-Alonso and colleagues strengthened the evidence through a multicenter clinical trial that involved 85 patients experiencing recurrent implantation failure (RIF) and 25 control participants [54]. The ERA’s capability to discern between receptive and non-receptive endometrial states led to more personalized embryo transfers (pET). Results were encouraging, RIF patients had pregnancy and implantation rates of 51.7% and 33.9% respectively, with even non-receptive patients showing increased rates when pET was adjusted according to ERA findings.

Another early study focused on the misalignment of the WOI among RIF patients, and similarly, employing ERA-guided pET improved pregnancy rates, highlighting potential diagnostic value of ERA in determining endometrial receptivity [55]. Confirming this, Hashimoto et al.’s research in Japan showed an increase in pregnancy rates following pET in patients with previously non-receptive endometria, reinforcing the value of personalized timing based on ERA results for successful reproductive outcomes [56].

A subsequent study by the initial ERA researchers reevaluated the test to refine the predictive transcriptomic sub-signatures within the WOI [57]. They developed a model to classify endometrium based on transcriptomic data, delineating four distinct WOI profiles, and linked them to different pregnancy success rates. An ‘optimal’ receptivity signature was associated with an 80% ongoing pregnancy rate for live births, while a ‘late receptive-stage’ profile indicated a heightened risk of unsuccessful pregnancy.

However, the narrative that ERA is a useful tool in the ART repertoire is not without contention. Emerging research has injected a dose of healthy skepticism, revealing no significant improvement—or in some cases, a decline—in pregnancy outcomes when ERA-guided protocols are employed. For instance, Neves and colleagues reported no notable improvements in pregnancy outcomes when applying ERA-guided protocols, with some evidence suggesting a potential decrease in success rates in oocyte donation groups [58]. Moreover, a study involving 248 patients with unexplained RIF found no significant differences in pregnancy outcomes when comparing patients classified by ERA as receptive or non-receptive [59].

Cohen et al. compared the ERA with traditional histologic dating in a study involving 97 patients with a history of implantation failure [60]. They found a concordance rate of only 40% between the two methods. Following the ERA-based pET, the clinical pregnancy rate was 26.7% for receptive patients and 22.5% for non-receptive, indicating no significant benefit of ERA-based personalization over standard protocols.

A large randomized clinical trial involving 767 patients who underwent single euploid FET concluded that using ERA did not result in improved live birth rates compared to the standard protocol [61]. The live birth rates did not differ significantly between the intervention and control groups, with 58.5% in the intervention group and 61.9% in the control group. Similarly, a recent a retrospective study of 861 women revealed no differences in outcomes between receptive and non-receptive patients as identified by ERA [62].

Comprehensive systematic reviews and meta-analyses by Liu et al., Arian et al., and Zolfaroli et al. have further evaluated ERA’s impact in IVF [63,64,65]. Liu et al.’s analysis of 11 studies suggested some benefit for RIF patients deemed non-receptive by ERA, but not for those with a generally good prognosis [63]. Arian et al. found no significant enhancements in live birth rates or ongoing pregnancies in a review of 8 studies involving 2784 patients [64]. Zolfaroli et al. echoed these findings with a large analysis of 12 studies and 14,224 patients, casting doubt on the clinical utility of ERA in enhancing IVF outcomes [65].

Recent studies have contrasted earlier overly optimistic findings regarding the ERA, challenging the initially assumed benefit. In line with these later studies, guideline bodies such as ESHRE do not endorse ERA. They state specifically, ‘While there are insufficient data to recommend the routine use of any commercially available test of endometrial receptivity to diagnose the cause of RIF, assessment of specific aspects of endometrial function by testing can be considered [66]’. This underscores the importance of thorough assessment and research before new technologies are adopted into clinical practice.

### 3.2. Other Endometrial Genetic Profiles

Beyond the ERA, the exploration of endometrial genetics has expanded through advances in ‘omics’ approaches. These new techniques have identified numerous gene profiles involved in the endometrial cycle that may have a predictive value in ART.

An initial study in this field by Kao et al. utilized advanced microarray technology to analyze the gene expression of 12,686 genes during the human endometrial window of implantation (WOI), identifying 156 significantly up-regulated and 377 down-regulated genes during this critical phase [67]. Complementing this, another study analyzed endometrial biopsies from fertile women, noting substantial expression changes in 211 genes from the pre-receptive to the receptive phase [68]. Horcajadas et al. expanded this list, confirming known gene involvement and recognizing new markers [69]. These early investigations offered new insight into potential markers for diagnosing endometrial receptivity with greater precision.

Haouzi et al. sought to discern genetic markers indicative of endometrial receptivity to enhance IVF success rates [70]. By conducting endometrial biopsies from women normally responding to fertility treatments across the early and mid-secretory phases, they identified five genes with marked activity increases during the latter phase, offering potential new biomarkers for embryo implantation readiness. Further research compared endometrial gene expression of women with unexplained infertility against fertile controls [71]. Through high-density arrays analyzing 44,000 genes, this study distinguished 145 genes with significantly higher expression and 115 with reduced expression in infertile women, implicating these genes in fertility and suggesting their utility in understanding infertility at a molecular level.

Focusing specifically on patients with RIF during IVF treatment, Koler and colleagues compared the gene profiles of endometrial samples from 12 fertile women and 20 RIF patients, taken on the 21st day of their menstrual cycle [72]. They identified 313 genes with altered expression in the RIF group, revealing down-regulation of genes critical for cellular processes and suggesting estrogen dependence in some pathways. Another investigation into RIF and recurrent miscarriages (RM) identified thousands of genes with differential expression [73]. Disruptions in immune response and nervous system development were seen in RIF, whereas RM affected organ and tissue development and the muscle system, underscoring the complex endometrial environment in these reproductive issues.

Several other studies have explored the endometrial transcriptome in RIF patients. Koot et al. established a gene expression signature that could predict RIF with excellent accuracy, achieving a PPV of 100% [74]. Choi Y and colleagues further examined both the uterine transcriptome and microRNAome in RIF patients, revealing disruptions in key signaling pathways [75]. Additional research identified 357 differentially expressed mRNAs in patients with RIF compared to successful IVF/ICSI attempts [76].

To explore whether the microRNA (miRNA) composition of endometrial secretions could predict IVF outcomes, researchers employed a non-invasive aspiration method to gather secretions from infertile women before ET [77]. The study found distinctive miRNA expression associated with varied IVF outcomes, suggesting these miRNAs as potential markers for implantation success. Long noncoding RNAs (lncRNAs) have also been evaluated as potential endometrial biomarkers for RIF. Xu et al. examined how lncRNAs interact with miRNAs and mRNAs, identifying differentially expressed lncRNAs through RNA sequencing [78]. Expanding on this, Chen et al. conducted genome-wide profiling to further understand the role of lncRNAs in women with RIF, identifying 1202 differentially expressed genes in these patients, which included 742 lncRNAs and 460 mRNAs, highlighting the regulatory roles of lncRNAs in the endometrial receptivity of women with RIF [79].

While significant advances in the field of endometrial ‘omics’ have improved our understanding of genetic and molecular markers during the implantation window, considerable gaps remain. These include the need for broader validation of genetic markers across diverse populations and their practical integration into clinical settings. The functional impacts and interactions of many identified genes and molecular pathways in fertility are still not fully comprehended. To address these challenges, future research must prioritize large-scale, longitudinal studies to validate these markers in real-world settings. Such efforts could bridge the gap between theoretical research and clinical application with the hope of enhancing the prediction and success rates of fertility treatments.

### 3.3. B-Cell Lymphoma 6 (BCL6)

The expression of B-cell lymphoma 6 (BCL6) protein, an inflammatory marker, has been a subject of interest in the field of reproductive medicine due to its overexpression in endometriosis and link to progesterone resistance [80,81]. Furthermore, a new commercial test, ReceptivaDx, has been developed to assess BCL6 levels in the endometrial lining.

Almquist et al. analyzed endometrial BCL6 expression’s predictive value for IVF outcomes, finding that low BCL6 levels were correlated with significantly higher clinical pregnancy and live birth rates [82]. In contrast, those with high BCL6 expression had substantially lower rates of success, indicating that high BCL6 is a strong predictor of poor IVF outcomes, notably in cases of unexplained infertility. Further research assessed IVF success following medical or surgical intervention for suspected endometriosis in women with abnormal BCL6 expression. Improved live birth rates were observed in those receiving medical suppression or laparoscopy compared to controls, underscoring the benefit of addressing aberrant BCL6 levels prior to IVF [83].

Expanding on these findings, Dan et al. studied the impact of endometriosis treatments—Lupron and laparoscopy—on IVF outcomes in patients with BCL6 overexpression as identified by the ReceptivaDx test [84]. Among the 143 women who tested positive for BCL6, those treated with Lupron had an 81% pregnancy rate and a 68% live birth rate, while laparoscopy treatment resulted in a 75% pregnancy rate and a 55% live birth rates. BCL6-negative women who did not receive treatment still experienced relatively high success rates, suggesting that endometriosis treatment could improve IVF outcomes in patients with high BCL6 levels. A retrospective analysis further supported BCL6’s predictive value for diagnosing endometriosis, finding a high positive predictive value of 96% for the test in detecting the condition [85]. Notably, some patients with BCL6 overexpression but no endometriosis presented with other inflammatory pelvic conditions, highlighting BCL6’s potential as a screening tool.

Moreover, a recent study evaluated how uterine preparation methods for FET influence BCL6 expression [86]. It revealed that BCL6 overexpression was less common in cycles using exogenous progesterone compared to natural cycles, indicating that the hormonal environment during uterine preparation could affect BCL6 levels and potentially IVF outcomes. However, contrasting findings emerged from Klimczak et al., who reported no significant correlation between BCL6 expression and live birth outcomes among normal responders to IVF, challenging the marker’s universality in predicting IVF success [87].

While abnormal BCL6 expression is linked to reduced IVF success and may indicate endometriosis, the efficacy of BCL6 testing as a prognostic tool might be more relevant to certain subgroups within the IVF population. Of note, data are still very limited, and the utility of BCL6 testing requires further investigation across various treatment protocols and patient demographics.

### 3.4. Immune Markers

Several studies have evaluated endometrial inflammatory markers in the setting of fertility care, observing associations between specific cytokines and immune cells with IVF outcomes. Boomsma et al. introduced cytokine profiling of endometrial secretions as a non-invasive method to analyze the endometrial environment [88]. This prospective cohort study of 210 women undergoing IVF revealed that levels of monocyte chemo-attractant protein-1 and IFN-γ-inducible 10 kDa protein were associated with implantation and that interleukin-1β and tumor necrosis factor (TNF)-α levels correlated with clinical pregnancy. Additionally, Mariee et al. investigated interleukin 15 (IL-15) and leukemia inhibitory factor (LIF) within the endometrial tissue of women experiencing RIF compared to fertile controls [89]. Their findings indicated lower LIF levels and heightened IL-15 levels in the RIF group, suggesting an altered endometrial expression pattern of these cytokines may play a role in implantation difficulties.

Delving into the correlation between IVF success and endometrial cytokine concentrations, another prospective study of 50 women undergoing IVF observed that successful pregnancies were often associated with lower levels of TNF-α, IP-10, and MCP [90]. This suggests that diminished levels of these cytokines might reflect an environment more conducive to implantation. Building on this, a study involving various fertility-challenged populations—including those with hydrosalpinx and unexplained infertility—showed that a higher count of CD56+ uterine natural killer cells and increased HLA-G expression were predictors of successful pregnancies [91]. In addition, the presence of HLA-F was positively associated with these cell counts, adding a layer to the immunological predictors of successful IVF. Another prospective study analyzed IL-1 and TNF-α levels in endometrial secretions from 76 women in their first IVF/ICSI cycle [92]. They reported a significantly higher concentration of IL-1 in the group that had successful chemical pregnancies compared to the group with failed pregnancies. However, there was no significant difference in clinical pregnancy outcomes.

A large prospective study involving 1738 patients looked at how endometrial immune profiling could personalize fertility treatment to enhance success rates [93]. Personalized treatment based on these endometrial immune profiles significantly improved pregnancy rates for infertile patients with RIF or RM, achieving 37.7% and 56% success respectively, compared to 26.9% and 24% in those patients with normal immune profiles (*p* < 0.001). Moreover, a recent study with 128 participants explored whether the distribution and density of endometrial immune cells could be prognostic of pregnancy outcomes following IVF and embryo transfer [94]. The results underscored that higher densities and clustering of uNK cells and macrophages correlated with IVF failures, proposing these immune cells as potential prognostic markers.

Diao and colleagues developed an endometrial immune cell-based score (EI score) to predict implantation success in IVF/ICSI patients [95]. The study, which included a derivation cohort of 139 couples and a validation cohort of 29, found significant differences in immune cell profiles between those who became pregnant and those who experienced implantation failure. EI scoring, based on these markers, was validated and showed a c-index of 0.82, indicating a strong predictive performance, and an accuracy rate of 79.3% in the validation cohort. The researchers concluded that EI scoring could assist clinicians in estimating the chances of successful implantation in IVF/ICSI treatments.

Together, these studies emphasize the complexity of immune regulation in the endometrium and highlight the potential of cytokine and immune cell profiling in developing personalized approaches for fertility treatments. Although some of these studies show promise, the available evidence is still limited. According to ESHRE, ‘peripheral NK cell testing is not recommended’ and ‘uterine NK cell testing is not recommended [66]’.

### 3.5. Other Potential Endometrial Biomarkers (P450, Integrins, Prostaglandins, VEG-F, PYB)

The research on endometrial biomarkers for predicting ART outcomes is expansive and continually evolving. Brosens et al. assessed the prognostic implications of endometrial aromatase P450 mRNA expression and noted its high levels corresponded to poor IVF outcomes, with significantly lower clinical pregnancy rates, positing a new prognostic tool for ART success [96]. The role of integrins, particularly the luminal expression of αvβ3, was also evaluated, revealing a significant correlation between positive luminal αvβ3 integrin expression and successful IVF-ICSI treatment [97]. Vivella et al. investigated the role of prostaglandins, particularly PGE2 and PGF2α, during embryo implantation, finding their levels peak during this period [98]. They posited that manipulating these prostaglandins or their receptors impacts embryo adhesion, indicating their levels as potential non-invasive biomarkers for endometrial receptivity.

The influence of oxidative stress biomarkers on IVF outcomes was also scrutinized. A cohort of 100 patients undergoing IVF for male infertility had endometrial secretions analyzed pre-ET [99]. Results showed a successful link between higher antioxidant levels and reduced lipid peroxidation with positive IVF outcomes, suggesting a conducive environment against oxidative stress for implantation. Seo et al. explored specific endometrial proteins’ expression and its connection to IVF success [100]. Higher levels of VEGF-A were observed in the early luteal endometrium of the pregnant group, suggesting its potential role as a predictive marker for successful IVF pregnancies.

Proteomic analysis by Azkargorta et al. of endometrial fluid from women undergoing IVF treatments revealed increased inflammatory states and impaired glucose metabolism in unsuccessful cycles, identifying PYGB as a differentially expressed protein potentially indicative of implantation success [101]. Furthermore, the role of EHD1 in endometrial function was investigated, especially concerning decidualization [102]. Elevated EHD1 expression was found in the mid-secretory endometrium of women with RIF compared to fertile controls, indicating its overexpression disrupted crucial decidualization proteins and therefore could be a diagnostic insight and therapeutic target for RIF.

Various endometrial biomarkers have been explored for their prognostic value in IVF outcomes, however, for many of these markers, the evidence is often limited to single studies, highlighting a need for more research to further evaluate their clinical utility.

### 3.6. Future Directives

In the arena of ART, omics-based approaches offer the potential for precision medicine and custom-tailored patient care. However, the journey of the ERA provided valuable insights, cautioning us to temper optimism with scientific validation, underscoring the importance of an evidence-based approach to integrating new technologies in clinical practice. As we move forward, it is vital to ensure that the nuanced signatures observed from genomics, proteomics, and the like, are not only insightful but also actionable in enhancing the personalization of implantation strategies. The excitement surrounding these advanced genomic tools must be balanced with a commitment to thorough research and validation. Only with this prudent and evidence-based approach can we responsibly utilize the full potential of ‘omics’ in tailoring implantation strategies to the individual’s unique biology.

## 4. Conclusions

This review underscores the potential role of ovarian and endometrial biomarkers, both traditional and possible emerging ones, in refining prognostication and treatment within ART. In addition to the well-established AMH and early follicular FSH, additional ovarian biomarkers such as GDF9, BMP15, connexin 43, and GC gene profiling have emerged, but their connection to IVF outcome prediction remains a subject of ongoing investigation. Of note, despite the compelling prospects of gene expression profiling, the practicality of their use in current clinical settings remains a present challenge, yet it may be soon overcome with ongoing scientific advancements. The integration of newer biomarkers into clinical practice necessitates a judicious interpretation and robust data to substantiate their predictive value.

Although the ERA was initially presented as a promising approach in ART, recent studies have cast doubt on its utility, urging caution in prematurely adopting certain technologies before appropriate validation has taken place. Beyond the ERA, research into the transcriptome, BCL6 and immune markers has sought to identify changes that could signify endometrial receptivity. While these studies have identified potential new markers, their clinical application has yet to be validated.

In summary, although novel biomarkers hold promise for enhancing individualized patient care, a measured and evidence-based approach is essential to ensure that the use of these emerging tools does not outpace the speed of clinical proof and validation (Table 1). Although the future of ART may indeed witness the maturation of these promising biomarkers with their eventual integration into routine practice, it is important to acknowledge that many of these technologies are presently far from ready for clinical use. Innovation needs to be balanced with clinical efficacy and evidence-based medicine to achieve the ultimate goal of delivering safe, effective, and personalized care to those embarking on the journey to parenthood using ART.

## Figures and Tables

**Table 1:** Strength of available evidence for each biomarker per the Discipline-Based Education Research (DBER) rubric.

Biomarker	Strength of Evidence		
	Limited	Moderate	Strong
AMH			X
FSH			X
GDF9/BMP15		X	
Connexin 43	X		
GC gene expression	X		
CA125	X		
Caspase	X		
BCL6	X		
PTEN	X		
ERA			X
Other endometrial gene profiles	X		
BCL6	X		
Immune markers	X		
P450	X		
Integrins	X		
Prostaglandins	X		
VEG-F	X		
PYB	X		

Legend: Limited (few peer-reviewed studies of limited scope), Moderate (a well-designed study of appropriate scope that has been replicated by at least on other similar study OR a few large-scale studies with similar results OR a moderate number of small-scale studies with general convergence but possibly with contradictory results; if the results are contradictory, more weight must be given to studies that reflect methodological advances or a more current understanding), Strong (numerous well-designed qualitative and/or quantitative studies, with high convergence of findings). DBER reports (2012) Box 1-1 p. 18 [103].
